# Fair trade governance: revisiting a framework to analyse challenges and opportunities for sustainable development towards a green economy

**DOI:** 10.1007/s43621-021-00063-6

**Published:** 2021-12-28

**Authors:** A. Cristina Ribeiro-Duthie, Fred Gale, Hannah Murphy-Gregory

**Affiliations:** 1 Politics and International Relations Program, UTAS-School of Social Sciences, University of Tasmania, Sandy Bay Campus, Private Bag 22, Hobart, TAS 7001 Australia; 2 Politics and International Relations Program, University of Tasmania, UTAS-Arts Building, L113, Newham Campus, Launceston, TAS 7050 Australia

**Keywords:** Fair trade, Ethical consumption, Sustainable production and consumption, Green economy, Analytical framework of governance, Sustainability governance

## Abstract

As a relatively new form of non-state governance, the fair trade movement presents an opportunity to promote sustainable production and consumption and hence social change. Global market demands and consumer engagement denote changes in social practices that have led governments to share decision-making processes with private sector and non-governmental organisations. In this context of change, it is important to consider not only whether new forms of governance weaken or strengthen states’ authority within the marketplace but also the extent to which they may allow for “green washing” instead of the green economy proposed by the United Nations Environmental Program. This study considers the fair trade of food production and consumption as a potential innovative model. In doing so it examines the existing general literature on governance, which highlights that decision-making processes tend to reproduce top-down approaches. While such practices may reproduce conventional hierarchies, it is worth questioning the potential of new forms of governance within global markets. This article builds on a sustainability governance analytical framework to deepen understandings of fair trade governance and its possible responses to the dilemmas of food production for ethical consumption and thus sustainable development in transnational relations. This research aims to contribute to the literature on improving compliance with global sustainability standards and through this, inform practices that allow for cooperation towards a green economy.

## Introduction

Analysis of fair trade initiatives around the world regarding food products is now well established in the literature [1–13]. The increasing reach of this alternative trade model is attributable to a range of factors, such as the growing acceptance of new forms of governance; innovative business structures; new organisational, ethical consumer, and political attitudes and behaviours and new market strategies. The fair trade movement is of major interest for our research given its approach to production and consumption within food systems explicitly aimed at tackling the economic, social and environmental dimensions that determine sustainable development. While the balance of these three dimensions within each fair trade organisation and fair trade standard is unclear, the goal of sustainable development and social responsibility are explicitly stated in the mission, vision, aims, and impact study reports of the main fair trade organisations [1, 12]. In a context of increasing consumer awareness about the effects of unsustainable production and consumption patterns and the attempts of the fair trade model to address environmental protection, social inequalities, work conditions, and human rights, this form of global sustainability governance has gained attention from private and public decision makers, consumers, and scholars.

Many studies have sought to examine whether and how efficient fair trade schemes can be at promoting sustainability aims. This article draws on previous contributions to the debate about global environmental and sustainability governance [1, 14–18] to understand how the fair trade governance structure is organised to comply with its stated aims and intended sustainability outcomes or outputs. To do so, we synthesise an analytical framework to assess the governance structure of major fair trade organisations to then highlight how the scheme could broaden its contribution to a greener economy.

Given the growth and reach of this form of nonstate global sustainability governance, it is important to scrutinize the scheme’s governance and practices to assess its shortcomings and benefits. This is in line with previous recognition that “private authority can potentially help rachet up standards toward higher quality” [15, p. 217]. However, it is also necessary to avoid greenwashing, or in this case *fairwashing,* by ensuring a “robust mechanism for watching the watchers” [15, p. 218], hence the solutions need to be sound and not mere façades. Certification of food products will be used as an example to analyse the fair trade scheme’s approach and strategies for good practice on global sustainability governance.

Agriculture and food systems are resource intensive sectors intricately linked to climate change, therefore “designing better food production and distribution systems given the scarcity of water and energy and their roles to run food systems” [12, p. 1000] is necessary to improve the existing models to address climate change risks. From Cadman’s perspective, “the many parties both creating, and being affected by environmental change, must find ways of collectively solving problems of universal nature” [16, p. 18]. Schemes such as fair trade may also benefit from improved metagovernance arrangements, with metagovernance interpreted as “the management of governance networks that involve multiple participants and/or components” [16, p. 6]. We therefore adopt and adapt an already tested analytical framework to evaluate the governance structures of FT organisations.

To ascertain their performance against proclaimed social responsibility and sustainable development targets, we compare and contrast how the distinct schemes of the three major organisations of the fair trade movement are structured, their rule making arrangements, decision-making processes, and mechanisms of compliance and accountability. The relevance of this type of assessment is that the attributes of individual global social movements may impact their capacity to shape global politics as outlined by Bennett [19] and their potential to change institutions and norms [17].

The interconnectedness of sustainable development and the green economy can be recognised in the declaration of the 2010 G20 Seoul Summit leaders [20], when they referred to “sustainable green growth” as part of “a strategy of quality development”. For that to occur, they proposed enabling clean energy efficiency technologies through policies and practices, including technical, knowledge and practice exchange. The fair trade model for sustainable food production and consumption has potential as one of those enablers of quality development.

As highlighted by Cashore [21], the policy-making role of the state has been increasingly shared with non-state actors. This reflects governmental recognition of the expertise of global sustainability organisations. Indeed, fair trade organisations have participated in consultative committees of the Joint FAO/WHO Food Standards Program to help develop food standards, guidelines, and codes of practice [17, p. 801]. Therefore, an examination of how these global sustainability initiatives run their governance systems may benefit policy-making and decision-making aims at greening the economy and avoiding the mistake of weakly interpreting sustainability that then constitutes it as an oxymoron [22]. The analytical framework applied in this study offers a tool for assessing standard setters’ governance legitimacy given that they influence global sustainability governance and policy making. And policy making is a crucial role of a green economy proposal. According to Lederer et al. [23], the green economy proposal relies more on state roles of policy making, while sustainable development depends on partnerships among nonstate and state actors [23]. However, both conceptions are not totally dissociated, given that the United Nations Environmental Programme (UNEP) defines the green economy as an approach that “seeks, in principle, to unite under a single banner the entire suite of economic policies and modes of economic analysis of relevance to sustainable development” [24, p. 15].

While constituting a type of private initiative, or social enterprise, the reported aims of fair trade organisations can align with progressive civil society values and interests. In the environmental context, this is a sphere where non-state actors or transnational organisations have created space and elaborated new roles due to the lack of preparedness or expertise of states to address environmental challenges [15, 16, 25–30]. Moreover, as Abbot and Snidal [28] note, other roles matter beyond environmental issues, including human rights, labour rights, and other socioeconomic demands not always promptly or efficiently addressed by states. The fair trade movement’s targets, missions and visions capture values including environment and other aspects of sustainable development since its inception 30 years ago. This is of note, despite the term sustainability not always being employed with a clear definition or rigor. Sustainable global production initiatives have challenged the capacity of states by setting the rules of sustainability, undertaking good practices, and operating new forms of governance [28]. The new forms of governance involve private authority, as identified by Green [15], which can help states and nonstate actors solve cooperation problems [15], especially those related to environmental challenges embodied within the phenomenon of the Anthropocene [31, 32]. These challenges impact not only industrial and corporate behaviour, but also individual attitudes related to the environment and consumerism [33]. Fair trade embraces and reproduces a meaningful sustainability discourse by transforming it into a practical form of global market governance comprised of innovative food production standards. These are based on environmental elements such as avoidance of pesticides, low carbon practices based on recycling, soil management and use of compost; on social elements such as priority to small farmers and family producers to access international markets, capacity building to negotiate and trade in a globalised trade system, training and capacity building of small farmers, good work, health, and safety conditions secured;, and economic elements such as upfront payment for produce and a guaranteed floor price above the market price. A possible overlap can be identified with the leading role of the United Nations Environment Programme (UNEP) too as that organisation has, since the Rio + 20 Conference (held in 1992), promoted a “green economy” envisaging the necessary steps to secure “human well-being and social equity, while significantly reducing environmental risks” [34, p. 31]. The innovative aspect of these initiatives has been to integrate economic policy into environmental needs and sustainability concepts with the aim of “greening” the economy via “virtuous cycles of progress and prosperity” [35, p. 1024].

However, given that, according to Bernstein and Van Der Ven [36], governance structures can mask power relations and require continuous scrutiny, we aim to assess the governance legitimacy of the major fair trade organisations delivering sustainable production and consumption of foods. We do this by adopting an analytical framework in the form of questions to allow for the combined assessment of governance—proposed and defined according to Cadman [17], and sustainability-complemented by definitions of the International Social and Environmental Accreditation and Labelling (ISEAL) Alliance codes of practices (2014 and 2018). The former was especially helpful to define some of the parameters of organisational responsibility. From these contributions, a matrix to analyse sustainability governance is presented and applied to mainstream fair trade organisations. This framework is also applicable to other forms of global sustainability governance. Through this methodological approach, the present study seeks to offer tools for the improvement of potential initiatives for greening the economy as new forms of governance may play a significant role in shaping state policy and decision making. This is so because, as remarked by Lederer et al. [23], the green economy emphasizes the role of governments and national bureaucracies.

Fair trade is not the only “new form of governance” to merge sustainable development and environmental concerns. Both sustainable development and green economy have common targets in the three dimensions of social, economic, and environmental development [37]. Brand [37] indicates the potential of the green economy to become the leading strategy in political discourse and that sustainable development is “an attempt to reconcile environmental problems with those of development” [24, p. 28]. There are a range of actors exerting ruling authority in world politics, including nongovernmental organisations, firms, social enterprises, transnational networks, and voluntary certification [15]. Independent of the terminology chosen, Elliot and Schlaepfer [27] find that the model originally “designed to study public policy processes can also be applied to a private policy process” [27, p. 648] as borders are crossed for a global sustainable development. In this context, fair trade organisations could be considered private actors with power to exercise ruling authority. Drawing on the theoretical work of Green [15], who argues that “the rule-making activities of private actors have autonomous effects on global environmental politics” [15, p. 209], fair trade governance within food systems is recognised as playing an important role for agricultural (and rural) development, while offering sustainability standards to remote localities in developing countries, even where states lack presence or expertise [23, 33, 38]. As such, fair trade practices and expertise can contribute to ‘greening’ the economy in a more effective way, as it attempts to translate into actions the dimensions of sustainability [37]. At the same time, fair trade organisations may rely on the role of the state to influence the multiple actors involved for their system to succeed, as we demonstrated in a previous systematic review and analysis [12, 33, 38]. According to Biermann et al. [39], it can be argued that the institutional framework currently in place is not enough to address the necessary changes for sustainable development related to agriculture and all its effects for food security, addressing inequalities, social inclusiveness, and environmental impacts, to name a few. D’Amato and Korhonen [40] point out that “sustainability narratives” such as circular economy, bioeconomy and green economy provide guidelines for important “sustainability transformations post-Covid-19” [40, p. 107].

This global need might be addressed by associative governance, where governments or state agencies form governing partnerships with NGOs or societal organisations [4]. An example of private-interest government is where governments allow firms or business associations to set codes of practice or self-regulate their activities in certain sectors. Often, this self-regulation will take the form of voluntary standards, such as fair trade. A common argument for this self-regulation is that firms know better than governments or other outsider groups and will be able to develop rules that achieve particular aims at less cost. However, the balance between costs and benefits of governance through association depends upon institutional arrangements, the capacities of the associations and the strength of the state [4]. Kern et al. [41] believe that “policy mixes are required to address” conventional “market failures” and their “negative environmental externalities” [41, p. 2] as a pathway to addressing global sustainability challenges. In this sense, fair trade expertise may facilitate decision making and policy making [38]. By scrutinising fair trade governance, we generate lessons that may assist governments to green the economy.

Regardless of the potential benefits of nonstate governance actors, Abbot and Snidal [28] remind us of that action in global governance demands attention be paid to the potential for paternalism and to accountability issues—particularly in regard to “northern NGO efforts to represent southern workers” [28, p. 18], for example. As such, it is important to understand what private or nonstate governance involves, how new forms of sustainability governance operate, the challenges involved, and the areas for improvement towards the green economy.

Below, our study provides a literature review of new forms of sustainability governance and its connections with the green economy. The framework of analysis to assess fair trade form of sustainability governance, a synthesis of the contributions of Tim Cadman and the ISEAL Codes of Good Practices, is explained in the methods section. The mainstream fair trade organisations are outlined in order to highlight the aspects related to standards and governance in food systems. Data from those organisations were collected from the organisations’ repository and webpages that contain latest updates. The information gathered is assessed via the analytical framework with the findings presented in the results and discussion section. In the final section, we draw out the future directions of this research.

## A literature review on new forms of sustainability governance and their potential role to greening the economy

According to Bevir [42]: “governance refers to processes of rule wherever they occur” [42, p. 2] and this may extend beyond states’ authority. When explaining governance, Peters [43, p. 6] remarks that “there has been a shift away from an authority-based style of governing that has assumed the capacity of governments to exercise hierarchical control over society”. This has involved a shift in the constellation of governing actors. In this context, private governance comprised of non-state actors has gained space and representation, but there remains a distinction between state-centred governmental processes and society-centred governance processes  [28, 29, 44]. Peters [45]  remarks that “the idea that national governments are the major actors in public policy and that they are able to influence the economy and society through their actions now appears to be in doubt” [45, p. 223]. Cashore  [21]  notes that a “market-oriented consumerism” is driving this change.

Traditional governance centred on the state to guide society by top-down solutions has been challenged by forms of governance that no longer rely solely upon the state and its institutions [45, 46]. Indeed, in the current context, “steering society towards common public goals” is shared with a proliferation of actors that can enact governance—among which we can place fair trade organisations. Recognising that the state is no longer the sole dominant actor regulating the externalities of production, an increasingly important innovative solution in transnational governance is a multi-actor response involving states, firms, and NGOs, especially in the case of regulatory standard setting (RSS) such as for the fair trade scheme [28]. Therefore, the use of the term government is lessened, and the use of the term governance (or steering) is more often employed [43]. Here, the argument is that there must be a way of addressing and implementing common objectives of social actors [43], and this would be framed as governance. As there are many diverse meanings of ‘governance’, a brief discussion of the nuances surrounding the term is now undertaken.

While government remains an important player in governance, Peters [43] explains that it is necessary for the government to become involved with other partners—giving rise to terms such as multistakeholder governance, network governance, public private governance partnerships and so forth—to attain more effective forms of rule. At the other end of the spectrum, however, there are those who consider government superfluous or even ineffective in certain circumstances [21]. This “governance without government” perspective, which has strong influence from European schools (UK and Netherlands), is explained by Peters [43] in the following terms: “society is presumed to be better able of understanding its own affairs and of finding remedies for any problems that are encountered in its functioning” [43, p. 15]. Analogous to schools of economic thought about the market governing itself, society is thought to have the capacity to process rules of social organisation without relying on government agency. In this light, fair trade can be viewed as an example of a solution originating from society’s need for self-organisation inside the marketplace and having to deal with its economic, social, and environmental externalities. However, the fair trade model requires adherence to a set of rules, sometimes quite stringent for food production, which differ from a free market [47]. In this context of governance change, discussion of whether non-state market driven governance weakens states or attends to its broad strategies [21, 48] is relevant, especially considering the United Nations Department of Economic and Social Affairs (UN DESA) call for a green transformation including waste reduction and sustainable farming [37]. If we consider the triangulation governance perspective from Abbot and Snidal [28], the multi-actor partnership would increase efficiency.

In this sense, the standards underpinning private governance schemes need to be regarded individually per product or by geographical location against a set of values—set by another actor such as an auditor—to assure quality. This is the certification procedure that mainstream fair trade organisations have adopted. In the area of food production, private governance schemes such as fair trade have produced positive as well as negative outcomes in different regions, countries, and localities across the world [6, 13, 47, 49–52]. As noted by Auld et al. [46], an important question is whether competing standards tend to produce a “‘ratcheting up’ effect, so that weak regimes are either driven out of business or are forced to strengthen their rules, or a ‘race to the bottom’ where private regimes weaken their rules to compete for adherents” [46]. Assessments to date have produced diverse results, which justifies the need for more applied research in relation to food production and consumption.

Green [15] observed that existing studies of entrepreneurial authority are limited and suggests that cross-case studies may better support the generalisability of findings from global environmental governance to other areas. Green’s theoretical and empirical contributions to global sustainability governance are insightful regarding fair trade, which resides in the category of “entrepreneurial authority” capable of “improving outcomes” [15, p. 223] within food systems. As observed by Brand [37], the fair trade movement shares aim in common with (or supplementary to) the green economy political strategies of UN DESA [37], for instance the proposal that prices should “reflect the internalization of external costs” to “encourage sustainable consumption” [37, p. 29] to “promote the greening of business and markets” [37, p. 29]. However, the analysis of governance practices and impact assessment studies regarding fair trade certification are small in number, and the majority are commissioned studies. Cadman [17] highlighted “the calls for researchers to look at institutional design in more creative ways” [17, p. 6], and he developed an analytical framework based on dynamic interactions inside organisations, especially those involving decision-making processes that we review in our analysis.

This article combines insights from Tim Cadman’s analytical framework [17, 29] with the ISEAL Codes of Good Practices for sustainability [53, 54] to assess the governance practices and legitimacy of fair trade organisations’ reported activities. Cadman [17] recommends cross-disciplinary innovative methods to analyse governance, and we believe this may contribute to the strength of new forms of global sustainability governance enabling a greener economy.

## Methodological approach

The analytical framework to analyse global sustainability governance proposed here—presented as a matrix—seeks to address the potential for collaboration and improvement for sustainable development across the different certification schemes selected rather than simply promoting competition among fair trade organisations. As outlined above and detailed below, this matrix was adapted from Cadman [17, 29] and ISEAL [53, 54]. In the case of multiple certification standards, such reflections on metagovernance arrangements are seen as providing quality assurance to reach common ground [55, 56]. Cadman’s and ISEAL’s perspectives on sustainability governance are outlined below.

### Cadman’s analytical framework

In the view of Cadman [17], assessing participation and deliberation within institutions involves analysing patterns of social-political interaction. Deliberation is a form of democratic interaction where cooperation and common agreement through rational discourse contribute to problem solving and facilitate effective decision-making [17, p. 5]. This is different from the traditional form of aggregative democracy, which tends to be competitive rather than interactive [17, 57–59]. This approach was proposed by Cadman as a basis to assess global governance forms.

According to the author, the arrangements for democratic participation can determine the level of institutional legitimacy of global governance arrangements. Cadman suggests that his analytical framework may be extended to other “market-based governance systems” [29, p. 607] such as fair trade to assess which models are most effective [29]. Previous robust research demonstrates that the decision-making processes of mainstream fair trade organisations deserve critical attention [2, 19, 27]. We adopt the categories proposed by Cadman [14, 17] to critically analyse the governance systems of fair trade organisations.

The Cadman analytical model derives from the author’s understanding of governance legitimacy, where “input legitimacy concerns itself with the structures and processes of governance, while output legitimacy is more interested in outputs and outcomes” [17, p. 10]. Additionally, the sociological approach to legitimacy stressed by Cadman allows for assessment of the quality of governance, which is related to the balance among three aspects: structure, process, and outcomes.

The social-political interactions within and across fair trade organisations related to food systems guide our assessment of this form of global sustainability governance. To do so, we adapt Cadman’s proposed four categories to three, as follows: Interest Representation; Accountability & Transparency; and Decision Making. The fourth category, “implementation”—defined as a series of steps to put commitments into practice [17]—will not be included in the present analysis. This is because the pathway to assess implementation would require impact assessment studies that are beyond the scope of the current investigation.

We supplement the Cadman analytical framework with the ISEAL indicators for accountability and transparency, given that “accountability within networks is a serious challenge in an environment of governing without government” [29, p. 50]. According to Cadman, governance structures need to be sufficiently sophisticated to address accountability at multiple levels [29, p. 51], whereas transparency is a precondition for accountability. Therefore, we employ the ISEAL Alliance’s transparency and accountability indicators in recognition of its metagovenrance role in assessing nonstate governance regarding sustainability.

### ISEAL code of practices

An undesirable effect of the competing number of standards governance arrangements designed to increase the level of stringency and generate a “race-to-the-top scenario” [60] is the potential exclusion of small operators that cannot afford the certification process or who produce insufficient quantities of a product to compensate for the cost of acquiring certification [56, 61–63]. Another undesirable outcome lies in the creation of divergent sustainability requirements, which may become a “nightmare for producers” [61, p. 803]. There are also concerns that multiple and competing certification schemes can produce fragmentation [55], threatening the credibility and legitimacy of the general approach to voluntary sustainability standards [64].

Given these critical perspectives, a common proposal to address the issues identified is to employ the metagovernance conceptualisation approach, which has been reconceptualised and extensively explored by Murphy-Gregory and Gale [16] to analyse the Forest Stewardship Council (FSC) and Fairtrade International. In addition to promoting convergence among private standards—and risking not achieving it because there are too many interests involved in standard setting or an “ideological commitment” [61, p. 804]—“meta-standardisation” assists individual organisations in the process of rule-making in transnational relations [61, p. 807]. Standard setters contribute to ongoing debates about how sustainability can be translated into concrete practices in the market and across sectors. In this sense, the expertise of global sustainability governance deserves further analysis.

Credibility challenges around sustainability standards have already been signalled by the ISEAL Alliance [44]. Other authors have recognised that the proliferation of sustainability or good practices standards “must be addressed in a meaningful way” [61, p. 804]. Moreover, as the Food and Agriculture Organisation of the United Nations recognises, the governance of transnational public goods such as food safety would benefit from “common grounds for assessing sustainability” standards [65, p. 6], which ISEAL has been undertaking. ISEAL is recognised as a “legitimizing agent” [36, 55, 61, 66] that may contribute to differentiating credible governance systems from others. As such, it is used in this study for our analysis of the governance of the major fair trade organisations that are accredited by ISEAL.

According to Bernstein and Van Der Ven [36], ISEAL is the “creator and disseminator of best practices for transnational sustainability standard-setters” [36, p. 535]. Furthermore, Loconto and Fouilleux [67] characterise “ISEAL’s institutional entrepreneurship as part of the institutionalization of the sustainability field” [67, p. 167]. Therefore, drawing from previous contributions [36, 55, 56, 61, 65, 67, 68], the ISEAL Codes of Good Practice [44, 45] complement and help define some governance aspects in addition to Cadman’s analytical matrix for assessing certification governance, especially those on organisational responsibility. An aspect we stress is that given the aims of sustainable development, food production under fair trade standards holds the potential to extend its benefits because it addresses the inequality of those most in need, i.e., small farmers from the global South who constitute the main population subjected to hunger in a global economy [65]. However, we are mindful, as already clarified by Gale and Haward [30], that the commodity power of Southern countries is very limited within a neoliberal context.

We highlight that the fair trade movement challenges conventional trade with the same market tools to address its negative externalities. In this sense, fair trade schemes are innovative entrepreneurial initiatives [38], whose rationales are clearly explained by Green [15, p. 218], who argues that understanding global environmental governance sheds light on global sustainability governance. Recognising fair trade as a global sustainability alternative for trade in line with new forms of governance—as per Abbot and Snidal’s [28] terminology—we will hitherto consider fair trade as a form of global sustainability governance.

While Green’s theoretical approach is based on global environmental governance, we see fair trade as a form of global sustainability governance, whose ends have been to manage socioeconomic and environmental problems caused by conventional trade. Green refers to “environmental problems” and “global environmental governance” [15, p. 2], while fair trade schemes focus on sustainability. In our previous study, we addressed the environmental aspects of the three dimensions of sustainability and argued for a more balanced tripartite model for sustainable development by the fair trade movement. In this article, we analyse the three main fair trade organisations and their governance structures to assess their proclaimed sustainability aims and potential contributions to a green economy. The ISEAL alliance defines itself as the global leader in “communicating what good practice looks like for sustainability standards” [54, p. 5], the strategy being, among others, to measure impacts. We relied on ISEAL’s guidelines to supplement the definitions of some parameters of sustainability, as presented in the next section.

From Cadman’s conceptions of legitimacy of governance [17, 29] complemented by some of the ISEAL [53, 54] indicators and definitions of sustainability, our analytical framework has been developed to compare three forms of global sustainability governance within the fair trade scheme related to food (see Table 1). The parameter “who provides the funds” was added to the indicator resources as a matter of clarification. The parameter of transparency was drawn from the ISEAL 2014 credibility principle as this definition was easier to operationalise.

**Table 1:** Matrix proposed to analyse global sustainability governance based on Cadman and ISEAL conceptions

Criterion	Indicator	Parameter	FT Organisation to be analysed		
			FI	WFTO	FT USA
Interest representation [Score 0 for No and 1 for Yes]	Inclusiveness [“participatory practices in place, how stakeholders/interested parties may participate/influence the decision making” ([29], p. 47)]	(A) All stakeholders may participate of decision making?			
		(B) How may stakeholders participate of decision making: agreement?			
	Equality [“the number of actors affected and their active participation, the weight, and the extent to which influence is equally distributed” ([29], p. 48)]	(C) Participation of members by geographical location? Does it include the 5 continents?			
		(D) How is the distribution by geographical location?			
		(E) How participation of small producers, advocacy groups? How are board members are distributed? Equally?			
		(F) A person = A vote?			
	Resources [“provisions to enable (producers) participation, such as money and expertise to allow for training that increase participants skills and confidence” ([29], p. 50–51)]	(G) Funds provided to small producers?			
		(H) Foreseeable conflict of interest on who provides the funds are reported?			
		(I) Training for capacity building in place?			
Organisational responsibility [Score 0 for No and 1 for Yes]	Accountability [“is a central aspect of the quality of governance” ([29], p. 50) “and is enabled by transparency” ([29], p. 51)]	(J) Information on how the system operates?			
		(K) Information on how the system is governed?			
		(L) Standards systems inform the development and content of the standard?			
		(M) Information on who is evaluated and under what process?			
		(N) Information on which ways stakeholders can engage? [53, p. 9]			
	Transparency [“public access to information and decision making procedures” ([29], p. 51)]	(O) Information is easily available? [53]			
		(P) How is information made available: free and online (this opposes information available only upon request) [53]?			
		(Q) Are there mechanisms in place to “encourage stakeholders, peers and the scientific community to scrutinise results and findings” [53, p. 23]?			
Decision making [Score 0 for No and 1 for Yes] *The parameter R (aggregative) is the only one in this matrix that has scores inverted, given that in the theoretical approach adopted, the best solution is a deliberative one*	Democracy [Score 0 for Yes and 1 for No]	(R) Aggregative?			
	Agreement [Score 0 for No and 1 for Yes]	(S) Deliberative?			
	Dispute settlement [Score 0 for No and 1 for Yes]	(T) Is there a mechanism in place when agreement is not reached?			

Source: Cadman [17, 29] and ISEAL [53, 54] adapted with respective definitions and score attributes for assessment added

The analytical framework adopted for our methodological approach allows for a systematic evaluation of how global governance is exercised by fair trade organisations. The aims are to assess the quality of governance and legitimacy of this growing global market and business model and understand how sustainability has been translated into food production and distribution on the ground.

By adopting a dual forced technique (a Yes or No approach) in the assessment of the governance of the organisations, our aim was to allow for a tangible result against the parameters indicated by Cadman combined with the ISEAL definitions, with less space for subjectivism. There is well-known criticism of the prominence of abstraction and multiple interpretations within sustainability scholarship and practice, hence allowing companies to obscure their degree of sustainability. The choice of the dual forced technique is of value in limiting pluralistic interpretations [61]. It provides a more objective method for assessing the legitimacy of the governance of the major fair trade organisations delivering sustainable production and consumption standards and has the advantage of being applicable to other forms of nonstate governance.

## The fair trade organisations selected to be analysed

The governance structures of the main fair trade organisations analysed in this study are briefly presented in the form of case studies below. Whenever possible, we prioritise the essential information referring to the adopted criteria for assessment via the indicators represented by the parameters in the matrix. After the three organisations are introduced, we schematically set out their governance data in Table 3 before providing the analysis. The information presented is based on data from webpages of the mainstream fair trade organisations.

### Fairtrade International (FI or FAIRTRADE)

Fairtrade International presents itself as part of a “global fair trade movement” [69, preamble] that sets standards for producers, workers, and trader organisations. The FI Constitution states the belief that “trade can be a fundamental driver of poverty reduction” for change towards “greater sustainable development” [69, preamble].

The setting of standards is the method adopted to achieve the constitutional purpose of Fairtrade International, which is to promote “sustainable production practices, sustainable development and trade” [69, § 2.4.1]. The standards serve as guides for farmers and traders in building their organisations to attain sustainable production practices [70].

The key guidance of the standards is meant to ensure that producers are paid prices that cover sustainable production; secure a ‘social premium’ that provides sustainable development of their communities; enable prefinancing for producers who need it; promote long-term trading partnerships; and set the criteria for production and trade according to fair social, economic, and environmental conditions. There are specific requirements for small-scale farmers and for hired labour as per principles listed in Table 2, and both should abide by the following common principles: social development; economic development; environmental development; and no forced or child labour.

**Table 2:** Principles set for the two types of producers from Fairtrade International

Principles for small-scale producers’ organisations:	Principles for hired labour force situations:
Most of the members have to be smallholders who do not hire workers, instead uses their own work or workforce from their families	Have a committee to manage the Fairtrade Premium
All members have a say in the decision-making processes of the organisation	Right to join workers union and negotiate their wages and working conditions
Enable small-producers to build and enhance strong producer organisations	Salaries must be equal or higher than the average for the same regional average. WHS conditions assured

Source: FI [70]

The institutional structure of the Fairtrade system is outlined in the General Assembly document of the organisation, and key decisions including revisions to the constitution are made in annual gatherings of representatives. The General Assembly is the highest authority of the organisation, where members exercise their responsibilities through delegates that represent them. Only national or regional fair trade organisations and producer networks are eligible for membership in the FI association, and only one member in the same territory from the same membership group is allowed. To achieve the ultimate aims of improving the livelihoods and working conditions of small-scale producers, the organisation relies upon its board. This board is primarily in charge of the strategic plan of the organization—which should be approved in the annual general assembly—and is also responsible for compliance with the standards and approval of official policies related to the scope of the association’s work “geographically or by product or production process” [69, § 8.2.2].

The board is constituted by 9 to 13 persons elected, appointed, or nominated by members, with three participants of the board serving as independent members. One member on the board is nominated, elected, or appointed from each continental network: Africa (FTA), Asia-Pacific (NAPP), and Latin America-Caribbean (CLAC). Each member of the Fairtrade Board undertakes a term of 3 years and may be re-elected and re-appointed for a second period. Members should declare any duty or interest that may conflict with the interests of the organisation, and the board may decide to withdraw any voting participation that is considered a conflict on a specific matter. Board members should “abide by all rules and by-laws of the Board”, including “a requirement to maintain confidentiality of all information entrusted to them as members of the Board during and after their term of office” [69, § 9.7]. Fairtrade Board members do not receive any remuneration.

The board responsibilities are to set committees that will oversee finances and secure budget for the organisation’s projects (i.e., the Finance and Audit Committee); the Governance Committee oversees possible improvements of the organisation’s structures and processes; and the Nomination Committee. In addition, the board appoints three bodies:

1. The Standards Setting body “for use by producer and trading” organisations [69, §2.2.1].
2. The supervisory board of FLO-CERT to oversee the quality, efficiency, and sustainability of the certification process.
3. Any other committee to deliver activities or functions for the promotion of sustainable development, as set at §2 of the same Constitution of the Fairtrade organisation.

Fairtrade International and the World Fair Trade Organisation are the two global networks that jointly lead the Fair Trade Movement. Together, they have released the Fair Trade International Charter, which replaces the 2009 Charter of Fair Trade Principles, and this new Charter works as a reference “to restate the fundamental values of Fair Trade that unite the diverse range of organisations and networks that make up the Global Fair Trade movement” (WFTO website, *International Fair Trade Charter*).

### World Fair Trade Organisation (WFTO)

WFTO focuses on both fair trade and social enterprises. Its certification system is based on the whole structure and business model of an enterprise and not on the commodities themselves. In other words, the WFTO mark certifies not a specific product or ingredient but the entire company—if the business puts “people and planet first” [71].

Defined as a global community of businesses “producing and trading, campaigning and educating for a better world” [69], WFTO was born in 1989, although its beginnings can be traced back to the 1940s, involving activists concerned with “farming and weaving, marching and lobbying, teaching and trading to take our vision forward” [72]. As per their statement, they seek “new models of business and trade that drive fair and sustainable economies” (WFTO website, *International Fair Trade Charter*) [73]. To obtain WFTO certification, enterprises should demonstrate that “they put people and planet first in everything they do” (WFTO website, *About us*). This can be translated into behaving according to their 10 established principles [74], as listed below:

1. opportunities for disadvantaged producers, i.e., poverty reduction through trade;
2. transparency and accountability required from all stakeholders;
3. fair trade practices;
4. fair payment and a fair price;
5. no child labour, no forced labour or human trafficking;
6. no discrimination, gender equity, freedom of association and equal pay;
7. good working conditions;
8. capacity building;
9. promote fair trade;
10. respect for the environment, which involves sustainable sourcing, production techniques, managing waste, purchasing policy, packaging, and shipping.

These 10 principles inform the WFTO standards [75] against which an enterprise’s management and operations are assessed to attain the *WFTO guarantee system*, which is at the heart of the WFTO certification. These standards constitute common fair trade values—such as those demonstrated in the International Fair Trade Charter; and are also informed by the International Labour Organization (ILO) conventions on human rights, among others. Some of them are mandatory from the outset of membership, while others should be accomplished according to an agreed timeline. Continuous improvement is enforced, and compliance of members is regularly assessed through self-assessment, peer visits and audits. In addition, there is a “Fair Trade Accountability Watch” where members can report the noncompliance of any of their certified enterprises or members.

The organisation is run democratically by members, including fair trade organisations (FTOs) that market fair trade products (producers or homeworkers), fair trade networks (FTNs), and fair trade support organisations (FTSOs). All producers, workers and members have participatory processes for their decision-making, including the representatives at the board, and this process is said to be in ‘continuous improvement’. The regional sites of WFTO—in Asia, Europe, Latin America, Middle East & Africa—should have representation on the board. Each regional site holds an Annual General Meeting (AGM) of members, has a Board of Directors, a Coordinator Office, and staff.

### Fair Trade USA (FTUSA)

Launched in 1998 by Paul Rice in the USA, the organisation was named Transfair USA and started by trading coffee. In 2010, it was renamed Fair Trade USA (FTUSA website, *Timeline*), for which Fair Trade Certified serves as the certifying body. The organisation is ruled by a Data Governance Policy, which is guided by the following principles: data management; confidentiality; data quality; data storage, access, and use. Moreover, a quality manual specifies how FTUSA operates to deliver their theory of change, which is defined as “a model where people prosper in resilient and sustainable communities through building a market for responsible business and mutually beneficial trade that cultivates conscious consumption” [40]. The quality manual details the “organisation’s structure, mode of operation, mission, values, and quality management system” [76].

FTUSA’s organisational structure comprises functional teams and category teams. The former is responsible for setting standards, assurance, and impact—in accordance with ISEAL good practices. The latter follows the requirements and procedures as per the FTUSA’s program assurance manual [77]. There are standards governing each FTUSA program, which include agriculture, trade, apparel and home goods, and seafood. The standards are said to ensure that all stakeholders’ views are considered in the decision-making process of FTUSA. Standards are made available to all stakeholders, and views or suggestions for improvement may be made through the organisation’s webpage.

A multi-stakeholder Board of Directors includes representatives from producer organisations, workers’ rights organisations, industry representatives, NGOs, funders, and technical experts. Board nominations can be made by existing board members, staff and/or stakeholders to ensure that the process of nomination is accessible. Decision-making is undertaken by consensus whenever possible, and if a consensus is not achieved, decision-making follows the Bylaws of the Board of Directors by voting where the winning vote equals 50% + 1. A single stakeholder group must not comprise more than 50% of the board. The board must approve any new standards or revisions. The board also oversees the strategies and finances of the organisation. In addition, the senior management team (SMT) is “collectively responsible for operations planning, business strategy development and execution, project prioritization, and quarterly business review” [77, p. 11]. The SMT includes executive leadership team (ELT) members, vice presidents and department directors. Finally, there are external parties who provide support services, such as field consultants, survey coordinators, and conformity assessment bodies (CABs), who provide on-site auditing services. CABs must adhere to the complete ISO 9001 lead auditor training (or a corresponding training); and to the ISO 19011:2011 Guidelines, among other requirements [78].

To ensure that FTUSA relations with their partners are objective, impartial, and independent, any conflicts of interest in the past, present or future are to be reported by any staff upon hire. All “FTUSA personnel sign a confidentiality agreement which requires that they not disclose trade secrets or confidential business information” [77, p. 12] from other parties or use this information unless the data are already publicly available.

According to the FTUSA website, in its 20 years of existence—from 1998 to 2018—the organisation has delivered “$551 million in cumulative financial benefit to producers, including nearly $380 million in Community Development Funds and more than $172 million as a result of the Fair Trade Minimum Price” (FTUSA website, *Timeline*). FTUSA considers the success of its core products as the driver to attract businesses and products to their model.

## Results and discussion

This study considers the case of fair trade (FT) within food systems and its potential impacts on natural resources. Fair trade schemes are prominent forms of new or private governance that, if well addressed and managed, can contribute not only to sustainable development but also to the greening of the economy. Throughout its development as a form of governance, the main fair trade organisations consistently refer to their aims about improving sustainable development such as addressing poverty, inequality, human rights, and working conditions. However, how do these new forms of governance deliver their proclaimed global sustainability standards?

From the case studies summarised, we see decisions on products to be included in the model are made democratically in the general assemblies, where key decisions are made. However, it is of note that Cadman has a stringent definition of democratic decision making, where decision by agreement (or “deliberative” decision making) is considered its best form. Cadman explains that in a context of environmental disruptions that affect all involved, the many parties “must find means of collectively solving problems of a universal nature” [29, p. 18]. Table 3 presents our assessment of the organisations’ performance.

**Table 3:** Findings from the three fair trade organisations analysed according to our proposed framework

Criterion	Indicator	Parameter	FT Organisation to be analysed		
			FI	WFTO	FT USA
Interest representation [Score 0 for No and 1 for Yes]	Inclusiveness	(A) All stakeholders may participate of decision making?	0	1	1
		(B) How may stakeholders participate of decision making: agreement?	0	1	1
	Equality	(C) Participation of members by geographical location? Does it include the 5 continents?	1	1	0
		(D) How is the distribution by geographical location?	1	1	0
		(E) How participation of small producers, advocacy groups; board members are distributed? Equally?	0	0	0
		(F) A person = A vote?	0.5	0.5	0.5
	Resources	(G) Funds provided to small producers?	1	1	1
		(H) Foreseeable conflict of interest on who provides the funds are reported?	0	0	0
		(I) Training for capacity building in place?	1	1	1
Organisational responsibility [Score 0 for No and 1 for Yes]	Accountability	(J) Information on how does the system operate?	1	1	1
		(K) Information on how is the system is governed?	1	1	1
		(L) Standards systems inform on the development and content of the standard?	1	1	1
		(M) Information on who is evaluated and under what process?	0	0	1
		(N) Information on which ways stakeholders can engage? [53, p. 9]	1	1	1
	Transparency	(O) Information is easily available? [53]	0.5	0.5	0.5
		(P) How are information made available: free and online (this opposes information available only upon request) [53]?	0.5	0.5	0.5
		(Q) Are there mechanisms in place to “encourage stakeholders, peers and the scientific community to scrutinise results and findings” [53, p. 23]?	0.5	0.5	0.5
Decision making [Score 0 for No and 1 for Yes] *The parameter aggregative (R) is the only one in this matrix that has scores inverted, given that in the theoretical approach adopted, the best solution is the deliberative (S)*	Democracy [Score 0 for Yes and 1 for No]	(R) Aggregative?	0	0	1
	Agreement	(S) Deliberative?	0	0	1
	Dispute settlement	(T) Is there a mechanism in place when agreement is not reached?	1	1	1
Total—sum of scores of each fair trade organisation analysed			11	13	14

Our analytical framework combined Cadman’s three categories with the ISEAL Code of Good Practices to compare and contrast the three selected fair trade organisations. For the three cases analysed, the following total scores were obtained by each organisation: FI = 11/20; WFTO = 13/20; FTUSA = 14/20. The higher the score, the better the fair trade organisations are fulfilling these governance attributes and therefore attaining legitimacy as defined by Cadman [14, 29] and ISEAL [53].

However, the assessment intended to be objective, does leave space for interpretation. For example, a zero score indicates that the answer to the question is ‘no’ based on current information provided, but it may also be that the relevant data to answer the question was not found despite an exhaustive search (webpages consulted for information are listed at a separate section in the references list). The most difficult question to answer based on current data was (E) as it was unclear from the information provided whether the groups within the three organisations are equally represented on the decision boards or more broadly within the organisations. Although it is recognised that all organisations analysed possess mechanisms to represent each group it cannot be concluded that the number of people from each group is “equally distributed”.

While global transnational voluntary standards seek to sidestep the Westphalian sovereignty that limits states, our question focuses attention on the actual capabilities of fair trade schemes for transnational enforcement of sustainable consumption standards and assurances of social change. In this context, the legitimacy of fair trade organisations is important, and scrutiny of fair trade governance arrangements should be expected. Among the indicators, inclusiveness appears to require attention by FI compared to the WFTO and FTUSA. Governance mechanisms targeted at including producers have long been discussed by scholars such as Bennet [25] and Raynolds [2]. From the documents analysed, not all stakeholders may participate in decision-making, and the governance processes involve nomination or vote but not always agreement. This has impacts on the distribution of participants.

The equality in participation by geographical location and group representation could not be attributed to FT USA, and the inclusion of the five continents was the condition to obtain the full score in this parameter. This aspect demonstrates that according to the matrix proposed in Table 1, if we analysed fair trade reports alone, which are heavy on rhetoric for addressing inequalities and inclusiveness, we would not capture these governance issues around interest representation. We note that among fair trade organisations, not all groups are equally represented, as Bennett [26] and Raynolds [2] have already demonstrated with vertical analysis of decision processes within fair trade organisations.

Regarding the resources provided for the autonomy of small farmers, this is not transparently informed but could be inferred. However, there was no clear report about conflicts of interest regarding the funds obtained by each organisation for that aim. Thus, while on parameter (G), we attributed the full score to all organisations, this did not transfer to parameter (H) given that conflicts of interest in relation to the funds obtained by each organisation were not reported.

Misrepresentation can have paternalism as a side effect, according to Abott and Snidal [28]. One way to avoid paternalism is by providing resources for capacity building and training of small farmers from remote rural settlements around the globe so they are better equipped to make choices. Supporting the autonomy of small farmers and at the same time enable them to exercise good environmental standards is a desirable sustainability practice. This role would traditionally be conceived as a government role, as Green [15] has demonstrated, but it can derive from combining private expertise and ruling authority. Nevertheless, the recognition of fair trade expertise in securing good practices in production, distribution and auditing its own processes has helped to boost the sustainability of their food production. Even so, following the parameters of our analytical framework, organisational responsibility also requires attention. Considering their accountability and transparency, further improvements are needed, as shown per their overall lower scores on these indicators. Thus, from our findings, fair trade organisations analysed should enforce their global sustainability governance regarding the parameters proposed in Table 1 tested by Table 3. In highlighting this finding, we are not aiming to create standards for standard-setters but conduct an analysis of fair trade governance. We have done so to understand the potential areas for improvements for boosting a global sustainability model of governance that has potential positive impacts on socioeconomic and environmental decisions, which contribute to a green economy. The last thirty years of the fair trade movement has proven the model’s resilience and degree of maturity that certainly endows the organisations to address the most critical issue identified in our analysis: the provision of information to secure greater transparency, i.e., provide “public access to information and decision making procedures” [29] p. 51]. This differs from redefining transparency or using it out of the context of governance to refer to the transparency of contracts and relations to producers, suppliers, or market partners, which are also relevant. Therefore, we utilised the ISEAL 2014 indicators of transparency to allow for more tangible analysis.

Following our matrix parameters, no fair trade organisation addressed transparency across all the parameters defined by Cadman and the ISEAL. FI provides information but some of this is only available upon request, which does not attend to the criterion of “easily” and “free” information available. WFTO also stands halfway, and in that case, we used 0.5 as a score for all organisations. Information provided only upon request, or no data provided at all even after request may be encouraging or not, it is open to interpretations and as there is ambiguity, we attribute half score to all organisations on question (Q). In contrast to the best outcomes in decision making, FTUSA could not obtain the full score in distribution of participation by geographical location, whereas the other two organisations were able to demonstrate this attribute.

Data showing that the organisation appeared to partially attend the parameter occurred for four of the totals of twenty parameters. The lack of available information to enable a clear response or assessment reinforces its lesser performance on the accountability and transparency indicators abovementioned and defined (as per Table 1). We could not assess via this matrix if there are underlying institutional constraints impeding access to some data that might be strategic to each organisation. As stressed by Bennet [25], fair trade sustainability standards are also political constructs. By choosing to analyse fair trade organisations’ governance where it departs from a specific definition of legitimacy, our approach seeks to highlight another perspective on the governance of the major fair trade organisations, which have the potential to play an important role in realising a greener economy.

## Conclusion

We recognise fair trade as a form of global sustainability governance that constitutes a private authority given its expertise to guide food consumption and production according to clear steps and procedures based on stringent sustainability standards. Fair trade standards are more easily available and analysed as fair trade organisations need to make small farmers, retailers, businesses, and auditors understand their requisites. However, the mechanisms in place behind the delivery of good sustainability practices, standards and choice of products are under-explored or analysed. The pathway for our response regarding the governance legitimacy of the fair trade organisations was undertaken considering Cadman’s analytical framework, and this was supplemented via ISEAL accreditation guidance. We believe that a movement that aims to promote sustainable development such as fair trade should offer an efficient alternative to conventional forms of governance for the production and consumption of foods. The shortcomings of the fair trade movement need to be recognised and addressed, so a meaningful contribution to greening the economy is attained.

While the fair trade movement is regarded as providing high standards for ethical production and consumption for small farmers, we expect their level of stringency to be extended to large business partners [1] and urge greater attention to their internal governance mechanisms to improve performance. The analytical framework proposed here has demonstrated that there is some room for improvement. Their level of expertise to deliver sustainable production and consumption standards for multiple actors is broadly recognised. However, their governance dynamics are still falling behind in terms of legitimacy.

Green [15] highlights the impact and authority of transnational private actors when they opt for applying sustainability policies to their entire supply chain. This is certainly the case for the fair trade model, which shows the potential for change as fair trade revenues and global reach are increasing. The fair trade model for sustainable food production and consumption can be considered one of the enablers of quality development for greening the economy. Therefore, the assessment of the governance of fair trade organisations undertaken pointing towards the need for improvements is of relevance, as this allows for increasing the resilience of the model that proposes an alternative to conventional practices to address inequalities in trade relations. The critical points signalled here aim to contribute to possible improvements rather than promoting one organisation over the other. We seek to underline the mechanisms of governance as they currently operate and show some new possibilities.

It should be noted that limitations of this study concern the data for analysis being extracted from fair trade organisations websites, which can be changed overtime. Another limitation is the ‘implementation’ criterion from Cadman matrix that we did not adopt due to lack of adequate or extensive indicators to assess behavioural change. Given that social change is one of the main flags promoted by the fair trade movement, this gap on the assessment of consumer and organisational behaviour should be addressed in future research as we believe that better frameworks to assess the attainment of social change are needed. In expanding this research agenda with other methodological approaches, such as interviews, we envisage contributing further to knowledge about sustainability transitions and the green economy. As it stands, findings and analysis undertaken in this article contribute towards the attainment of the green economy strategies (as per UN DESA) which are in common with fair trade aims, thus offering support to decision making and policy making related to sustainable production and consumption at both state and nonstate levels.

## Data Availability

All data generated or analysed during this study are included in this published article (and the supplementary websites informed in the section two of the references list).
